# A Treatise on Malarial Fevers, for Domestic Use

**Published:** 1889-02

**Authors:** 


### Book Notices.

A Treatise on Malarial Fevers, for Domestic Use. By J. M. Lewis, M. D., of Mexia, Texas. Published by Eugene Von Boeckmann, Book and Job Printer, Austin, Texas.

This is a very nice little book about the size of a blue back speller. It contains ninety-six pages and is intended for "domestic use." The author advises chloral to be given in doses of from fifteen to twenty grains. This dose is rather large for indiscriminate use by the laity. He does not believe that calomel is indicated in any of the forms of malarial fever and thinks the patent medicine men display good judgment in eschewing it in their nostrums. The author thinks well of Simmon's Liver Regulator as a cathartic in malarial cases.

Take it altogether the book is, perhaps, as well suited to its object as any other of the kind extant.—B.

